# *G.3.2* is a novel allele of the gene *connector enhancer of ksr* ( *cnk* ) in *Drosophila melanogaster*

**DOI:** 10.17912/micropub.biology.001290

**Published:** 2024-09-26

**Authors:** Hanadi Chammout, Delia L. Adkins, Aleece K. Al-Olimat, Zeinab Alsaad, Beatrice M. Altopp, Tuqa Amer, Feyi O. Apampa, Gwendolyn R. Avery, Isaac I. Bazzi, Emilia D. Beck, Elise L. Beier, B. Shafer Belisle, Lane Benton, Madison M. Bolyard, Olivia E. Brain, Eldon T. Buckner, Shria Roy Chowdhury, Jennifer R. Cifranic, Liam Cleary, Tyler R. Clum, Autumn M. Cruz, Meghan V. DeGray, Isabel L. Echeverry, Haya El dana, Sarah K. Elkadri, Paige L. Estep, Luke R. Falke, Hannah J. Foor, Anika S. Gullapalli, Sandro S. Hakim, Hussein B. Hazime, Lauren E. Heininger, Emma G. Hoeft, Lauren M. James, Yeowon Jeon, Megan R. Johnson, Laine P. Jordan, Zayd Khan, Sydney K. Kochensparger, Fadi J. Koria, Ruby M. Krasnow, Veronica Lilly, Eileen Lim, Ian T. MacCormack, Andriy Malesh, Mikayla G. Mariano, Audrey C. Mentzer, Katelyn H. Messner, Katlyn C. Myers, Emily R. Newman, Annie M. Richters, Liliana Romero, Adam Rotem, Reese J. Saho, Kaname Sawaki, Ashley N. Selders, Elizabeth Shockney, Farah A. Sobh, Isabelle F. Speiser, Breanna M. Sproul, Veronica J. Sroufe, Antonia Tollkuci, Cassandra C. Trevino, Megan A. Vapenik, Erin M. Wagner, Kayla L Bieser, Jamie L. Siders, Justin R. Thackeray, Jacob D. Kagey

**Affiliations:** 1 University of Detroit Mercy, Detroit, Michigan, United States; 2 Ohio Northern University, Ada, Ohio, United States; 3 Clark University, Worcester, Massachusetts, United States; 4 Nevada State University, Henderson, Nevada, United States

## Abstract

Genetic screens in *Drosophila melanogaster* have long been used to identify genes found in a variety of developmental, cellular, and behavioral processes. Here we describe the characterization and mapping of a mutation identified in a conditional screen for genetic regulators of cell growth and cell division. Within a Flp/FRT system, mutant *G.3.2* results in a reduction of mutant tissue and a rough eye phenotype. We find that *G.3.2* maps to the gene *cnk* , providing further support that *cnk* is a critical gene in *Drosophila* eye development. This mutant was characterized, mapped and sequenced by undergraduate students within the Fly-CURE consortium.

**Figure 1. Characterization and mapping of the G.3.2 mutation as a novel allele of cnk .**
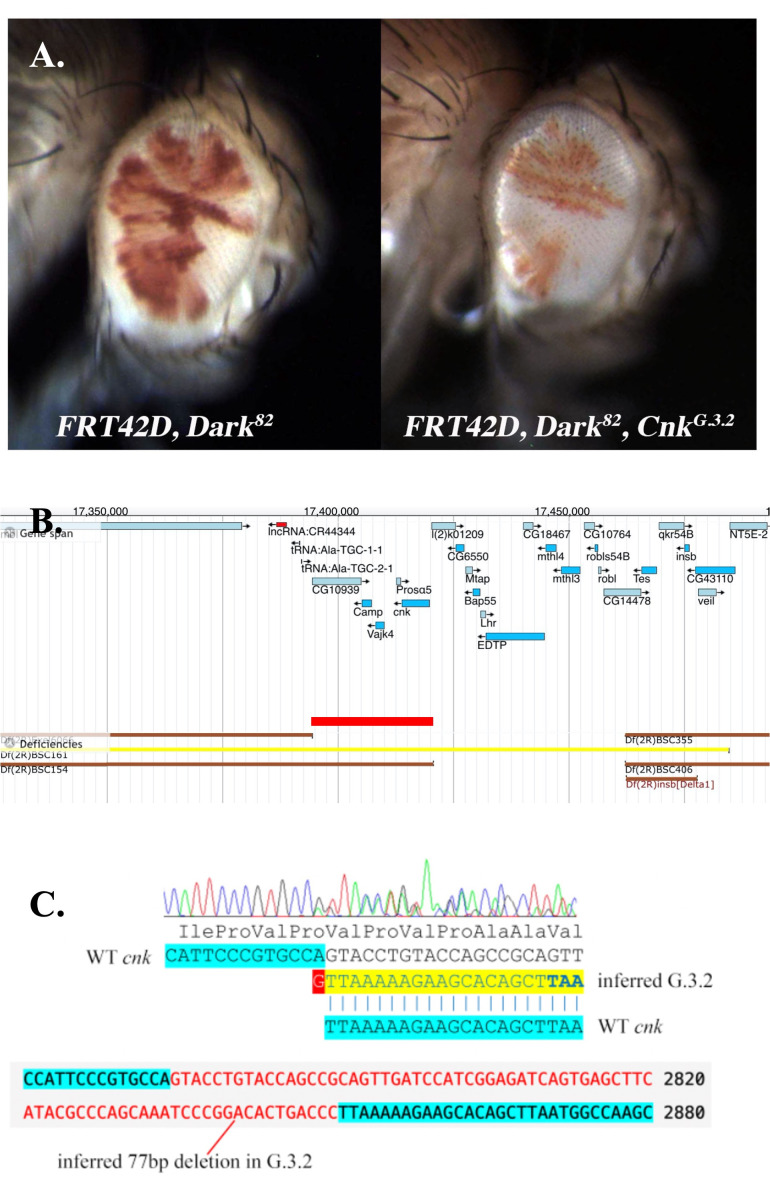
A). Comparison of control mosaic ( *FRT42D, Dark ^82^* ) and *G.3.2* mosaic ( *FRT42D, Dark ^82^ , cnk ^G.3.2^* ). In both panels the homozygous mutant tissue is *mw* +. B). Genetic map of region that failed to complement after mating *G.3.2* to the 2R deficiency kit. *BSC161* is represented in yellow, and failed to complement *G.3.2* and resulted in a region of 2R:17,097,569..17,462,347, red bar represents smallest overlapping area of failure to complement. C). Sanger sequencing of *G.3.2* and *Dark ^82^* , identifying a net loss of 76 base pairs within the *cnk* locus, identifying *G.3.2* as a novel allele of *cnk* , *cnk ^G.3.2^* . The chromatogram for this region is shown above our interpretation of the combined sequences; beneath that is a segment of the FASTA output for *cnk* within FlyBase, indicating the inferred 77 bp deletion in red. This deleted material is replaced within the G.3.2 chromosome by a single G, which is highlighted in red within the chromatogram interpretation; the resulting frameshift within G.3.2 results in a premature stop codon TAA, which is indicated in bold.

## Description

*Drosophila melanogaster* is a commonly used model organism that is an excellent tool for genetic research (Tolwinski 2017) . Genetic model systems of this type are particularly amenable to genetic screens, which provide an unbiased mechanism to identify novel genes associated with many different pathways (St Johnston 2002) . Here we report on the mapping and characterization of a novel mutation ( *G.3.2* ) isolated from a Flp/FRT EMS screen in the adult *Drosophila* eye (Kagey *et al.* 2012). This screen utilized an allele of *Dark* ( *Dark ^82^* ) on chromosome 2R, prior to mutagenesis (Akdemir *et al.* 2006), ensuring that there was a block in the canonical apoptotic pathway within the homozygous mutant mosaic cells. The overarching hypothesis from this screen was that there is a class of mutants that would have been missed in the first generation of Flp/FRT screens due to the residual apoptosis in the background. Previous mutants from this screen have been characterized and mapped by undergraduate students that are part of the Fly-CURE consortium, where students conduct this research within the structure of an undergraduate laboratory course (Merkle *et al.* 2023).

*G.3.2* was isolated from the Flp/FRT screen described above. Students from the University of Detroit Mercy, Ohio Northern University, and Clark University collaborated to characterize and map this mutant as part of the Fly-CURE consortium. Mosaic *G.3.2* eyes and control ( *Dark ^82^* ) mosaic eyes were generated through crosses of *♀ Ey-Flp; FRT42D* (BDSC 5616) mated to *♂ FRT42D, Dark ^82^ , G.3.2/CyO* or *FRT42D, Dark ^82^ /CyO,* respectively *.* Straight-winged F1 progeny with mosaic eyes were submerged in 70% ethanol and visualized at 40x magnification. The *Dark ^82^* ratio of red to white tissue was in alignment with previously published mutants from this screen [6]. Images were taken with AM Scope (AM 550). We find that the *Dark ^82^* mosaic eye is comprised of ~60% homozygous mutant cells ( *mw ^+^* ) while the *Dark ^82^ , G.3.2* mosaic eye has less mutant tissue (~30%) and results in smaller rough eye ( Figure 1A, *mw ^+^* is mutant tissue in both images). This suggests that the *G.3.2* mutation introduces a growth/development deficiency into the mosaic eye phenotype.

To identify the location of the *G.3.2* mutation we utilized the homozygous lethal nature of the mutation to conduct deficiency mapping with the 2R deficiency kit from the Bloomington *Drosophila* Stock Center (Cook *et al.* 2012). In parallel, unmated *♀* from *FRT42D, Dark ^82^ , G.3.2/CyO* were mated to *♂* from *Df(2R)/CyO* . This resulted in 86 deficiency crosses covering the region between the FRT42D location and the distal end of chromosome 2R. Complementation was scored if at least 10 straight-winged F1 progeny emerged, while a failure to complement was scored by reaching 100 curly winged flies without the presence of any straight winged flies. Any crosses that did not produce enough F1 progeny to determine complementation were repeated. All complementation testing was done in independent replicates by the students at each institution. From these experiments we find that mutant *G.3.2* failed to complement *Df(2R)BSC161* while complementing the overlapping stocks of *Df(2R)Exel6066* and *Df(2R)BSC355* ( Figure 1B ; see table 1 for breakpoints and full genotypes of deficiency stocks). These results identify the non-complementing region as 2R:17,097,569..17,462,347 ( Figure 1B ) (Ozturk-Colak *et al.* 2024). To narrow this region down further we used additional complementation crosses with deficiencies and single-gene lethal alleles (see table 1 for genotypes). From these experiments we found that *G.3.2* fails to complement a previously characterized lethal allele of the gene *cnk, cnk ^E-2083^* , indicating that *G.3.2* is a novel allele of *cnk* that we now therefore refer to as *cnk ^G.3.2^* (Therrien *et al.* 1998; Therrien *et al.* 1999).

Next, we utilized Sanger Sequencing to identify the specific lesion in *cnk ^G.3.2^* that is driving the mutant phenotype. Genomic DNA was isolated from single, heterozygous *FRT42D,Dark ^82^ cnk ^G.3.2^ /CyO* and *FRT42D,Dark ^82^ /CyO* adults using the method described by Gloor et al. (Gloor *et al.* 1993). A 5.9 kb genomic region encompassing the *cnk* open reading frame was amplified in seven overlapping reactions, each around 1kb long; these overlapping regions included all introns and about 500 bp upstream of the initiating methionine and 500 bp downstream of the stop codon. Sanger sequencing of each PCR product was carried out using three internal primers, all reading in a 5’ to 3’ direction (Psomagen). The chromatogram from one reaction transitioned to double-peaks about 200 nucleotides into the read, suggesting an indel event had occurred at that point that made the sequence from the balancer chromosome different from the *G.3.2* -bearing chromosome. After reading both bases in each double peak the CyO (wildtype for *cnk* ) sequence was subtracted, revealing the inferred sequence of the *G.3.2* chromosome. This contains a 77 bp deletion of *cnk* that removes bases 2,774-2850, combined with a single base insertion of a G ( Figure 1C ). This predicts a frame-shifting loss of 76 nucleotides, as shown in figure 1C, causing a truncation of *cnk* after P671, and a premature stop codon after the addition of six novel amino acids. This removes more than half of the *cnk* amino acid sequence, including the PH domain, and is therefore very likely to be a null allele. The sequencing data further supports our genetic mapping data, thus solidifying *G.3.2* as a novel allele of *cnk, cnk ^G.3.2^* . Further confirmation of *G.3.2* as an allele of *cnk* could be completed by utilizing rescue experiments with a wild type *cnk* allele.

Cnk (Connector enhancer of KSR) is a multi-domain containing scaffold protein identified as a positive regulator of the RAS pathway and plays a crucial role in the signaling pathway in *Drosophila* (Therrien *et al.* 1999). Previous studies have shown that an eye entirely mutant for *cnk* prohibits normal eye development in a similar manner to that observed in loss-of-function mutations in *Ras1* or *ksr* . Our data, showing a reduction in mutant mosaic tissue representation, further supports this finding and suggests the role in eye development of *cnk* is autonomous in nature. Additionally, *cnk* disruption has been shown to impact pathways downstream of Ras including Egfr signaling. Previously, the Fly-CURE has isolated an allele of *Egfr* from this same screen ( *Egfr ^L.3.1^* ). The *Egfr* allele and *cnk* alleles had similar mosaic phenotypes suggesting a similar disruption of *Drosophila* eye development (Stamm *et al.* 2019).

## Reagents

**Table:** 

Stocks from BDSC 2R Deficiency Kit				
Genotype	**BDSC #**		**Region**	**Complementation Results**
*Df(2R)BSC161*	9596	2R:17304783..17484828		Failure to complement
*Df(2R)Exel6066*	7548	2R:17097569..17394642		Complement
*Df(2R)BSC355*	24379	2R:17462347..17536673		Complement
Additional Deficiency stocks from BDSC				
*Df(2R)BSC154*	9541	2R:17304783..17420811		Failure to complement
Single genes tested for complementation				
Gene	**BDSC**	**Allele**		**Complementation Results**
*camp*	17063	* camp ^ep2499^*		Complement
*CG10939*	12034	* CG10939 ^06373^*		Complement
*cnk*	6577	* cnk ^E-2083^*		Failure to complement
*Bap55*	21174	* Bap55 ^EY15967^*		Complement
*l(2)k01209*	63391	* l(2)k01209 ^0540-G4^*		Complement
*mthl3*	84533	* TI{RFP[3xP3.cUa]=TI}mthl3 ^attP^*		Complement
*robl*	28484	* robl ^G7617^*		Complement
